# High salinity tolerance of invasive blue catfish suggests potential for further range expansion in the Chesapeake Bay region

**DOI:** 10.1371/journal.pone.0224770

**Published:** 2019-11-05

**Authors:** Vaskar Nepal, Mary C. Fabrizio

**Affiliations:** Virginia Institute of Marine Science, William & Mary, Gloucester Point, Virginia, United States of America; Australian Bureau of Agricultural and Resource Economics and Sciences, AUSTRALIA

## Abstract

In estuaries, salinity is believed to limit the colonization of brackish water habitats by freshwater species. Blue catfish *Ictalurus furcatus*, recognized as a freshwater species, is an invasive species in tidal rivers of the Chesapeake Bay. Salinity tolerance of this species, though likely to determine its potential range expansion and dispersal in estuarine habitats, is not well-known. To address this issue, we subjected blue catfish to a short-term salinity tolerance experiment and found that this species tolerates salinities higher than most freshwater fishes and that larger blue catfish tolerate elevated salinities for longer periods compared with smaller individuals. Our results are supported by spatially extensive, long-term fisheries surveys in the Chesapeake Bay region, which revealed a gradual (1975–2017) down-estuary range expansion of blue catfish from tidal freshwater areas to habitats exceeding 10 psu [practical salinity units] and that large blue catfish (> 200 mm fork length) occur in salinities greater than 10 psu in Chesapeake Bay tributaries. Habitat suitability predictions based on our laboratory results indicate that blue catfish can use brackish habitats to colonize new river systems, particularly during wet months when salinity decreases throughout the tidal rivers of the Chesapeake Bay.

## Introduction

Salinity tolerance is an important determinant of the survival, growth, reproduction and, ultimately, spatial distribution of animals in estuaries [1,2]. Tolerance of salt concentrations greater than those of its tissues depends on an organism’s ability to maintain its internal ionic concentration and to compensate for the loss of water from tissues [3,4]. However, freshwater fishes vary in their salinity tolerances; some are stenohaline and do not survive in salinities > 5 psu [practical salinity units]), whereas other species are euryhaline and can survive in salinities in excess of full strength seawater (34 psu; [5]). Understanding the salinity tolerance of freshwater fishes introduced in estuarine or coastal systems is crucial to determine their potential expansion and dispersal into novel estuarine habitats. For example, Prussian carp (*Carassius gibelio*), believed to be stenohaline, were introduced into small lakes in Estonia but subsequently spread along the entire Estonian Baltic coastline [6].

**Fig 1:**
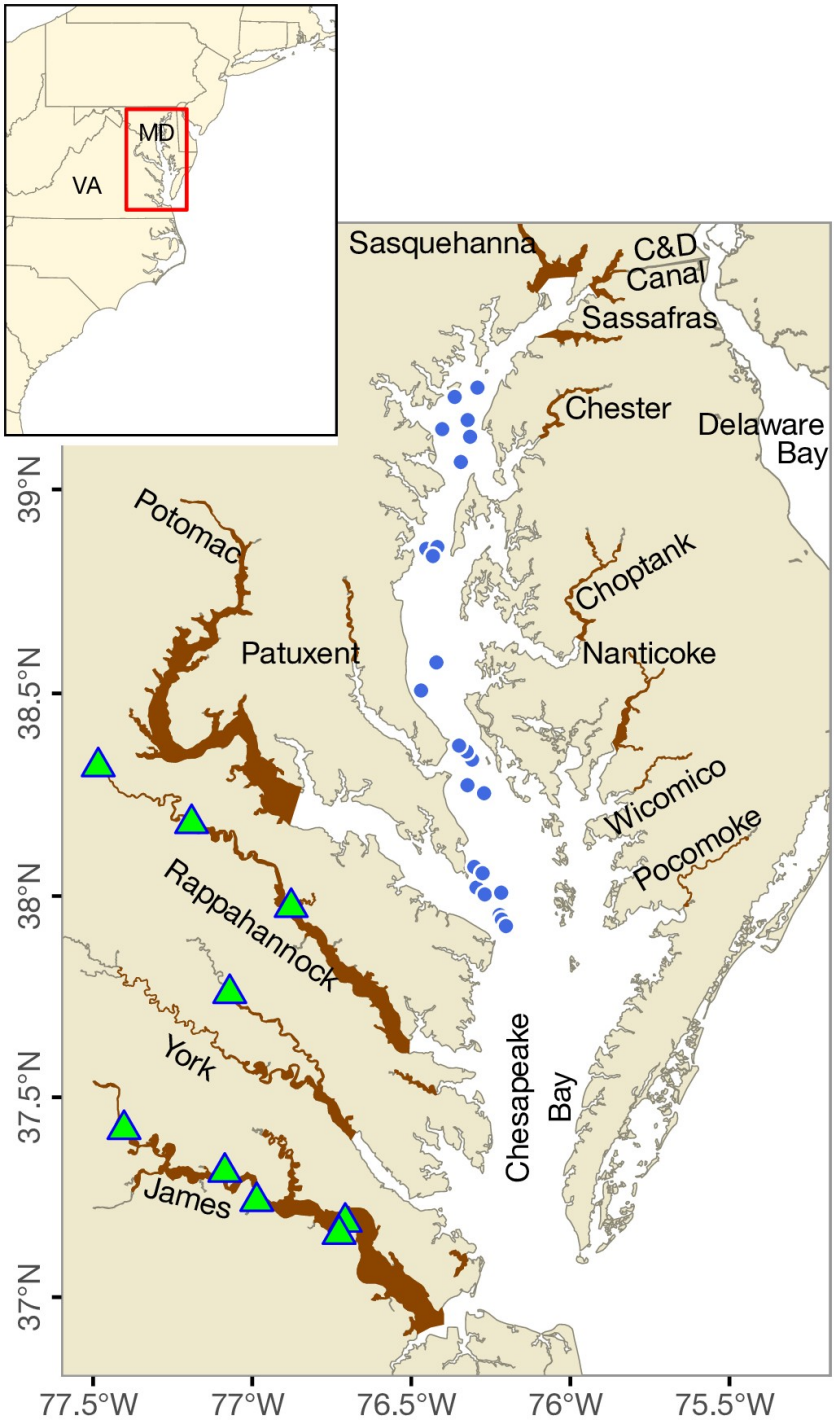
Stocking locations (green triangles, ▲) and current distribution of non-native blue catfish in Chesapeake Bay. Blue dots (●) correspond to additional locations where blue catfish were collected from the mainstem of the Chesapeake Bay during 2018 and 2019 (total fish collected = 63). The Chesapeake and Delaware Canal (C&D Canal) connecting the Chesapeake Bay with the Delaware Bay is also shown. Note that blue catfish have not been recorded from the Delaware Bay yet. Inset shows the location of the Chesapeake Bay in relation to Virginia (VA) and Maryland (MD). Figure available in color online.

Blue catfish (*Ictalurus furcatus*), a freshwater species native to the Mississippi, Missouri and Ohio River basins, have been introduced throughout North America due to their appeal to recreational fishers [7]. This includes the introduction to tidal freshwater regions of the Rappahannock, York and James rivers (subestuaries of the Chesapeake Bay) during the 1970s and 1980s, with a goal of enhancing recreational fisheries [8]. Blue catfish abundance subsequently increased and there are now an estimated 544 fish/ha, with an estimated population size of 1.6 million fish in a 12-km stretch of the James River [9]. Blue catfish have also expanded into tidal oligohaline (salinity < 5 psu) and mesohaline (5–18 psu) habitats throughout Chesapeake Bay (Fig 1; [8,10]) where they feed on vegetation, molluscs, and fishes [11]. They also compete with native species, such as white catfish (*Ameiurus catus*), whose population densities have declined concurrent with the increase in abundance of blue catfish [8].

**Fig 2:**
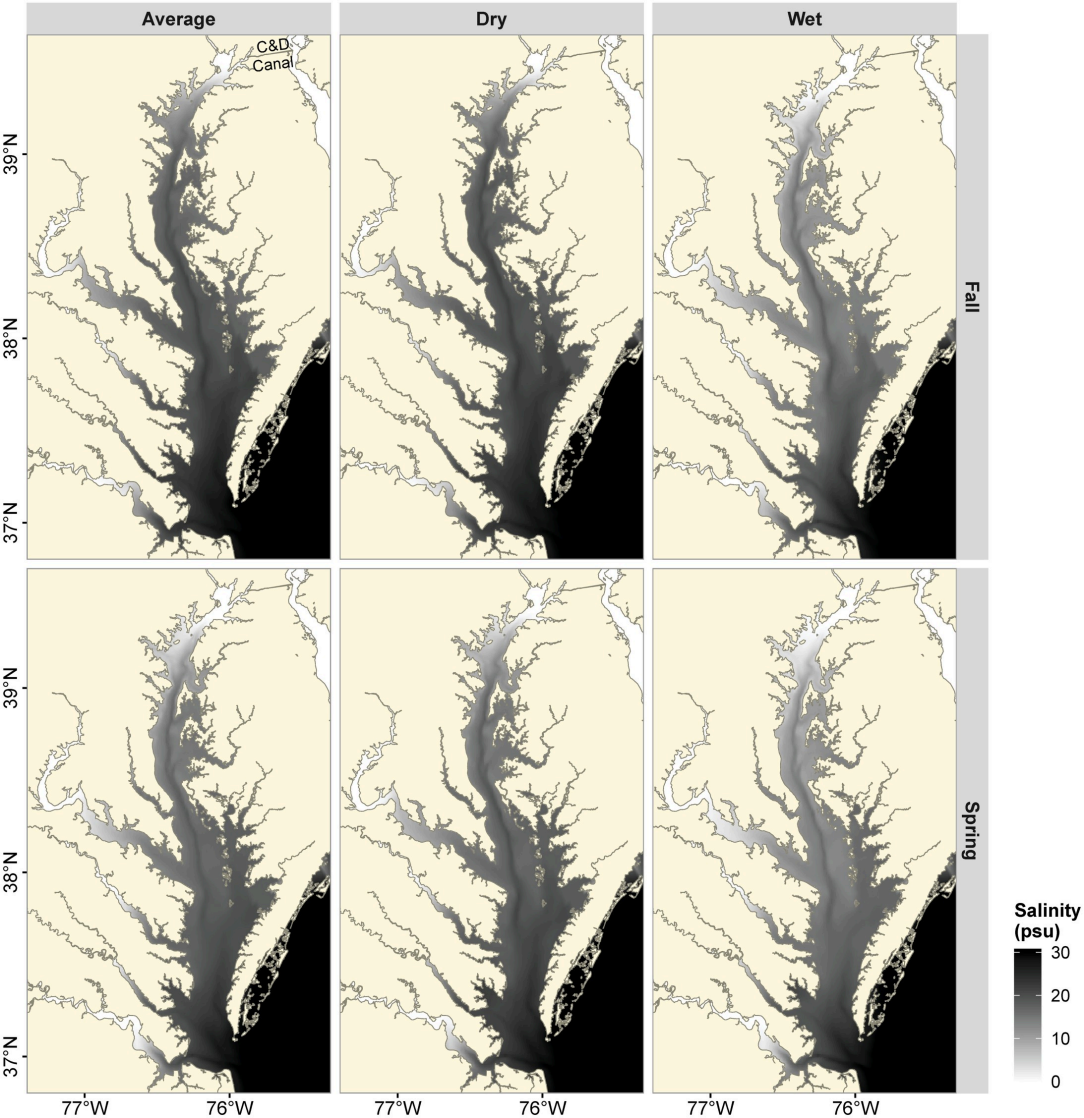
Vertically-averaged salinity (in psu) in the Chesapeake Bay typically encountered in spring (April) and fall (October) during average (2012), dry (2009) and wet (2011) years. Maps are based on the model developed by Du and Shen (2015).

The expansion and ultimate geographic range of blue catfish in the Chesapeake Bay region may be limited by their salinity tolerance. Salinities below 9 psu are not likely to affect the homeostasis of freshwater fishes like blue catfish because osmolality of the extracellular body fluids in freshwater teleost fishes is ~300 mOsm kg^−1^, which is isosmotic to this salinity [12]. If blue catfish are, however, able to tolerate salinities > 9 psu, then they have the potential to occupy and exploit a large fraction of habitats in Chesapeake Bay subestuaries, and thus, to negatively affect the ecological integrity of this estuary. A broad salinity tolerance presents an evolutionary advantage favoring spatial expansion in a climate of rapidly fluctuating salinity [12], as predicted for the Chesapeake Bay region in the coming century [13]. Current projections of the potential distribution of blue catfish in this region use a 14 psu tolerance threshold [10], which is the threshold reported for hatchery-reared blue catfish in their native range [14]. Yet, wild blue catfish have been observed in nearshore coastal waters of the southeastern U.S. (P. Fuller, USGS, *pers*. *comm*.) and in salinities up to 21.8 psu in the Chesapeake Bay [9]. Salinity in the mainstem of Chesapeake Bay varies seasonally and spatially from 0 to 28 psu due to > 5-fold differences in river discharge between dry and wet months (Fig 2; [15,16]). This might allow continued range expansion of blue catfish throughout the Maryland and Virginia portions of the Chesapeake Bay. In particular, the Chesapeake and Delaware Canal (“the Canal” hereafter) connects the upper Chesapeake Bay with Delaware Bay. If blue catfish are able to exploit the salinities in the Canal, they may also colonize Delaware Bay, potentially leading to negative impacts on estuarine resources and function in Delaware Bay.

Fisheries surveys and field measurements can indicate the salinity tolerance of blue catfish in the wild [17], though they cannot provide a causal link [18]. Observation of a species at a particular salinity implies that the species can survive at that salinity, at least for a short time, and information on the size, sex and other individual characteristics of the fish may be helpful in understanding differential tolerances of the species. However, the estimated salinity tolerance based on survey data may be biased because of a spatial and/or temporal mismatch of survey effort and fish distribution [17]. Particularly, for a range-expanding non-native species, the maximum field salinity where a fish is captured may be lower than the potential maximum, which may not have been realized. We contend, therefore, that laboratory experiments are needed to obtain a comprehensive and accurate characterization of salinity tolerance of blue catfish.

A robust measure of salinity tolerance using standardized laboratory methods has yet to be reported for blue catfish. Salinity tolerance may be assessed by exposing fish to increased salinities and recording the median lethal concentration (LC_50_, the concentration at which 50% of the individuals die within the specified period of time). It is, however, useful to understand how long a fish can survive at a given salinity, as quantified by time-to-death or survival models. These models, in turn, allow testing of the hypothesis that blue catfish can survive in mesohaline habitats long enough to allow movement between lower salinity environments. On the other hand, understanding how long a blue catfish can survive at a given salinity allows inferences as to whether this fish can use estuarine corridors as “saline bridges” to colonize rivers other than the ones into which they were originally stocked [19].

We assessed the salinity tolerance of blue catfish in a 72-hour laboratory experiment and compared the results with monthly survey data (collected over a 40-year period) from the Rappahannock, York and James rivers. We hypothesized that blue catfish will exhibit a relatively high salinity tolerance, allowing the species to expand in range throughout the Chesapeake Bay region. Our aim was to predict the suitability of different Chesapeake Bay habitats to illustrate potential colonization routes of blue catfish.

## Methods

All animal capture, handling, and experimental procedures were approved by the College of William & Mary Institutional Animal Care and Use Committee (protocols: IACUC-2017-05-22-12111-tdtuck and IACUC-2016-08-19-11376-mcfabr) and followed all applicable U.S. laws and regulations.

### Distribution of blue catfish in Chesapeake Bay subestuaries

To monitor the distribution of invasive blue catfish throughout its invasion history in the Rappahannock, York and James rivers, we used catch records covering 1975–2017 from the Virginia Institute of Marine Science (VIMS) juvenile fish trawl survey (conducted using a 9.14-m otter trawl towed along the bottom for five minutes). Temperature and salinity data (measured 1 m from the sea floor) were also collected. The juvenile fish trawl survey uses a stratified random sampling design between river kilometer (rkm) 64.4 and the mouth (rkm 0) of the Rappahannock, York and James rivers [16]. Survey stratification is based on depth and longitudinal region to ensure broad spatial coverage. As such, each subestuary is partitioned along its axis into four regions of ~10 longitudinal minutes and into four depth strata in each region (1.2–3.7 m, 3.7–9.1 m, 9.1–12.8 m, and >12.8 m). These areas are characterized by a gradient in salinity, which varies at both tidal and seasonal time scales [15,16]. In addition, salinity in the Rappahannock River is generally lower than that in the James and York rivers [16]. At each sampling location, all blue catfish were counted and a representative sample was measured (to the nearest mm). The gear used for the trawl survey is effective at capturing blue catfish ranging from 70 mm to 300 mm fork length (FL), although fish as large as 1000 mm have been captured [16].

To characterize changes in size structure of blue catfish along the salinity gradient, we used quantile regression [20], which better reflects salinity limitations on blue catfish than does mean regression [21]. The quantile regression approach also allowed for unequal variance in size of fish; such variance may arise from complex interactions of physiological limits and the invasion history of blue catfish. To characterize the effects of salinity on the minimum size of blue catfish captured in these subestuaries, we used piecewise quantile regression splines with conditional quantiles, *τ*, at 0.1 and 0.01. The quantile regression splines at conditional quantile *τ* = 0.1 and *τ* = 0.01 mean that about 10% and 1%, respectively, of the captured blue catfish were less than a given size at a given salinity. In addition, we fit regression splines with the 0.50 conditional quantile (*τ* = 0.5) to assess consistency in observed patterns for fish of median size as well. The salinities at which major changes in size structure of blue catfish occur can be identified using break-points (knots), which are values of the predictor variable (in this case, salinity) at which the adjacent polynomial splines are joined [20]. Two break-points were chosen for each subestuary based on the analysis of residuals. Quantile regression splines were fit in the statistical software R 3.5.1 using the package ‘quantreg’ version 5.38.

### Salinity tolerance experiments

Blue catfish were captured from the tidal portion of the James River (coordinates 37°14’N 76°52’W; salinity < 2 psu), and individuals between 165 mm and 265 mm FL were transported to the VIMS Seawater Research Laboratory. We selected fish between 165 mm and 265 mm FL for this study because these individuals are abundant in Chesapeake Bay subestuaries, and are readily captured by the trawl survey. At this size range, there is little sexual dimorphism in size-at-age of blue catfish such that both male and female blue catfish are between 1 and 3 years old (V. Nepal, unpublished data). Further, young of the year blue catfish (< 165 mm FL) are not likely to contribute to dispersal and range expansion of this population [9]. Fish were held for two weeks at 22°C at 2 psu salinity prior to use in an experiment. We used 2 psu for acclimation because preliminary experiments showed that blue catfish held in freshwater (0 psu) exhibited low survival rates and disease.

Based on a pre-trial study (see below), we chose 0 (control), 10, 13, 16 and 19 psu as the salinity treatment levels for the salinity trials, with 3 experimental aquaria (replicates) for each salinity level and 10 fish per replicate (total 150 fish). We used a gradual acclimation scheme (as opposed to a direct transfer of blue catfish to the target salinity) because the gradual acclimation resembles what blue catfish experience in the wild and has been reported to yield better estimates of field salinity distribution [17]. Ten random blue catfish were placed in each of the eighteen 340-liter circular experimental aquaria containing water at 0.1 psu and fitted with an air bubbler. All experimental aquaria were partially covered to reduce distress on the fish; the experiment was conducted using a natural dark/light cycle. Salinity was gradually increased by adding brine solution (55 psu) created by mixing Instant Ocean^®^ (Spectrum Brands, Blacksburg, Virginia) with filtered York River water (~18 psu) at rates such that the target salinity was reached in seven hours. The rate of increase in salinity we employed reflects that occurring due to tidal cycles in the section of the James River where the experimental fish were collected. All experimental aquaria were supplied water from the same brine solution mixed with de-chlorinated municipal water. Once the target salinity was reached, we monitored fish mortality by assessing reflex impairment every hour for the first four hours, then every four hours thereafter. Specifically, if a fish was unable to maintain equilibrium when handled, and exhibited reduced swimming ability or mouth gaping, the fish was considered moribund [22]. Moribund fish were immediately removed from the trial and euthanized by immersion in an ice slurry as recommended by Blessing et al. [23]. Five blue catfish died before meeting the above criteria for euthanasia (i.e. they had died during the periods between the four-hour monitoring checks). An additional fish that had jumped out of the tank at an unknown time was discovered dead later on. This individual was not used in the statistical analysis. The trials ran for 72 hours after the target salinities were reached. All fish that were alive at the end of the trials (n = 111) were euthanized.

**Fig 3:**
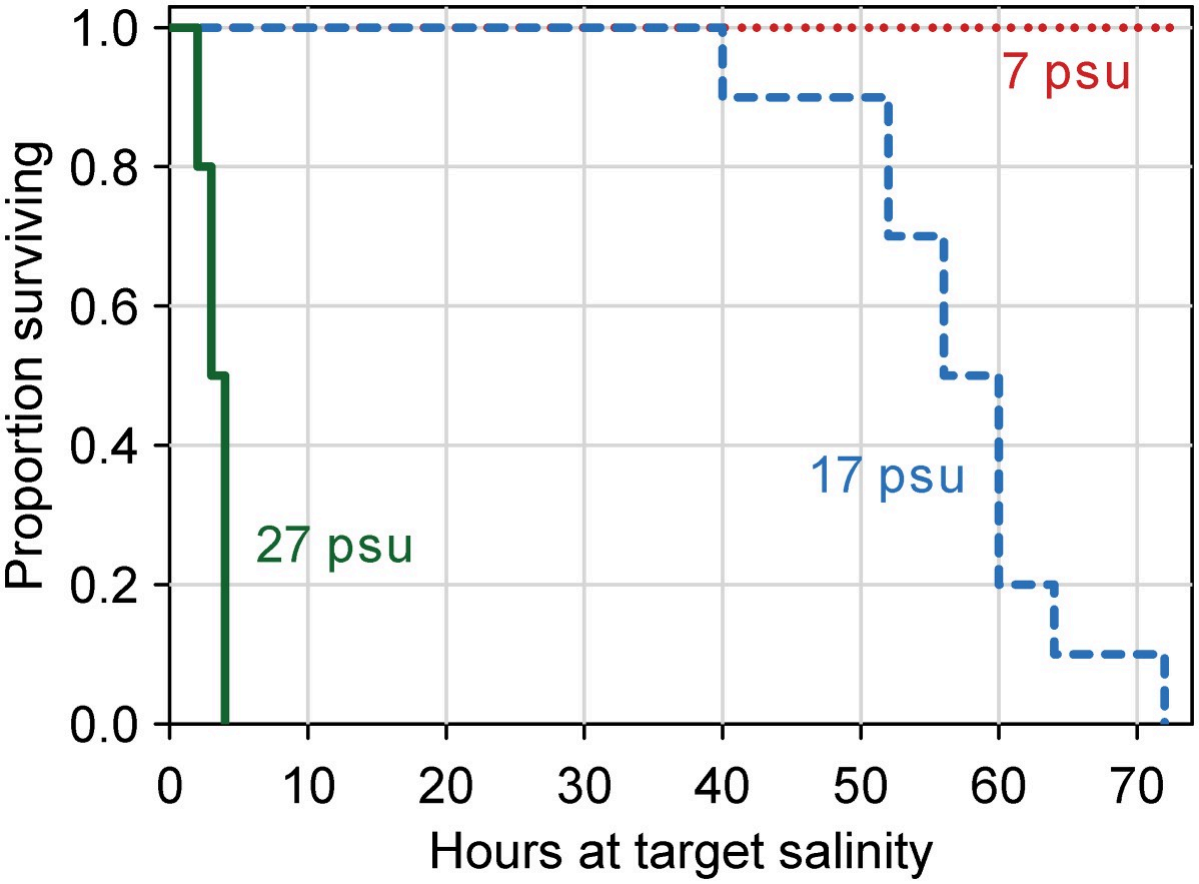
Survival of blue catfish over time after gradual transfer from freshwater to one of three salinity treatments during the pilot experiment. Figure available in color online.

To identify informative salinity levels for the salinity tolerance experiment, we performed a 72-hr pre-trial study, in which 10 fish were exposed to 7, 17 and 27 psu (total of 3 aquaria and 30 fish) at 22°C using the protocol described above. Fish were randomly assigned to treatment aquaria, though care was taken to ensure that the length ranges of fish were similar in all experimental aquaria. All individuals at 7 psu survived until the end of the experiment (72 hours), and all individuals at 27 psu died within 4 hours of reaching the target salinity (Fig 3). Mortality in the 17 psu treatment was first observed 40 hours after the target salinity was reached, and the last individual died was found dead 72 hours after the target salinity was reached (Fig 3). Hence, we chose 0, 10, 13, 16 and 19 psu as the salinity treatments for the main experiment.

To reduce the production of nitrogenous wastes, fish were not fed during the salinity trials. Water temperature, dissolved oxygen (DO), pH and ammonia concentration were measured daily, and 40% of the water was changed in each aquarium daily to maintain water quality. Water temperature ranged between 21.3 to 23.1°C (grand mean for initial and main trials = 21.9, S.E. = 0.1); DO ranged between 7.0 and 11.2 mgL^-1^ (grand mean = 9.1, S.E. = 0.2). Similarly, pH ranged between 7.6 and 8.2 mgL^-1^ (grand mean = 8.0, S.E. < 0.1), and ammonia ranged between 0.15 and 0.5 mgL^-1^ (grand mean = 0.35, S.E. < 0.1). Salinity was monitored to the nearest 0.1 psu using a handheld meter every hour during the 7-hour salinity increase period and twice a day thereafter. At the end of the experiment or upon death, fish were measured (mm FL) and dissected to obtain eviscerated weight (g). We subsequently calculated Fulton’s K as an index of body condition:

$$K\;=\;{W\times L^{-3}\times 10}^{5}$$ (1)

where *W* is eviscerated weight and *L* is the length of the fish. Sex of each blue catfish was assessed by macroscopic examination of the gonads.

To compare whether mean size and body condition of blue catfish were significantly different among the different salinity treatments, we combined observations from the pre-trial and the salinity experiment and fit linear mixed-effects models that modeled FL and body condition as a function of salinity and aquarium. The model took the form

$$y_{ij}\;=\;\mu +\;\beta Salinity+Aquarium_{j}+\varepsilon_{ij}$$ (2)

where *y*_*ij*_ is the response variable (FL or body condition) for fish *i* in aquarium *j*, *μ* is the overall mean FL or body condition, *β* is the rate of change in *y* with respect to salinity, *Aquarium*_*j*_ refers to the random effect of aquarium, accounting for potential similarities in observations among multiple blue catfish from a given aquarium (i.e., accounting for pseudoreplication), and *ε*_*ij*_ is the unexplained random variance in *y*. For each response variable, we fit a null model without salinity, and compared the likelihoods of the full and null models using Bayesian Information Criterion (BIC, [24]) calculated for each model as *BIC* = −2 * ln(*likelihood*) + *p* * *ln*(*n*), where *p* is the number of parameters in the fitted model and *n* is the sample size. In this approach, models with lower BIC values or with higher BIC weights represent the more parsimonious fit to the data [24].

Next, we modeled the effects of salinity, FL, sex, and condition on two responses: time-to-death and fate of the fish (i.e., whether it survived to the end of the experiment or not). We used a Cox proportional hazards model [25] to analyze the time-to-death and identify factors associated with changes in the risk of death. Potential predictors in the model included salinity, FL, body condition and sex.

$$h(t,\;X)\;=\;h_{0}(t)e^{\beta_{1}Salinity+\;\beta_{2}FL+\;\beta_{3}K}$$ (3)

where *h*(*t*, *X*) is the hazard rate at time *t* with covariates *X* (salinity, FL, condition [K] and sex of the fish), *h*_0_(*t*) is the baseline hazard function describing the change in risk of death per unit time at the baseline level of covariates (i.e., set at zero), and the *β*s correspond to the log-hazard ratio for the effect of each covariate on survival, adjusting for the other covariates in the model. Preliminary analysis indicated that stratification by sex was necessary to address the difference in baseline hazard rates between the male and female blue catfish (i.e., to address the violation of the proportional hazards assumption of the Cox model by the variable sex). Therefore, we obtained two baseline hazard functions—one for each sex [*h*_0*female*_ (*t*) *and h*_0_*male* (*t*)]. For the Cox model, we estimated robust standard errors for each parameter following Lin and Wei [26]; this approach accounts for potential similarities among individuals within each aquarium.

The fate of each fish (dead/alive) was modeled using a logistic regression model, with salinity, FL, body condition and sex as potential predictors.

$$ln(\frac{p_{i}}{1-p_{i}})\;=\;\mu \;\text{+}\;\beta_{1}Salinity+\beta_{2}FL\;\;+\;\beta_{3}K_{\;}{+\;\beta }_{4}Sex$$ (4)

where *p*_*i*_ is the probability of death for fish *i*, $ln\;\left(\frac{p_{i}}{1-p_{i}}\right)$ is the log-odds of death, μ is the overall mean log-odds of death, and *β*s are partial regression coefficients. Collinearity among predictors was checked graphically, and found to be absent (i.e., we found no evidence for linear relationships among pairs of predictors). Salinity was highly predictive such that all individuals exposed to salinities ≥ 17 psu died and all individuals exposed to salinities ≤ 13 psu survived. To avoid biases in parameter estimates and their standard errors caused by such quasi-complete separation, we used Firth’s penalized-likelihood logistic regression [27]. Currently, Firth’s logistic regression approach is limited to fixed effects, and thus, we included aquarium as a fixed effect in the model. As before, we calculated BIC and BIC weights for competing models and compared these metrics to inform model selection. Models within 2 BIC units of the best model were averaged using model weights [24], and the averaged model was used to estimate the 72-hour LC_50_ (salinity at which 50% of the individuals die within 72 hours). To permit comparison with previous studies, we also calculated LC_50_ using the modified Spearman-Karber method [28]. These calculations used the log of the doses, and as recommended by Hamilton et al. [28], 10% of the extreme observations were trimmed from each end of the response. We used R packages ‘lme4’ version 1.1–21 to fit linear mixed effects models, ‘survival’ version 2.43–3 to fit time-to-death models, ‘brglm’ version 0.6.2 to fit Firth’s logistic regression, and ‘drc’ version 3.0–1 for trimmed Spearman-Karber LC_50_.

### Spatially-explicit habitat suitability in the Chesapeake Bay

Model-averaged parameters from the logistic regression analysis were used to estimate the 72-hour survival probability of a blue catfish of size 224 mm FL for salinities between 0 and 32 psu. We selected 224 mm FL because this was the median length of blue catfish used in the salinity trials. Salinity conditions were based on the model by Du and Shen [15], which provides monthly mean salinity profiles (from surface to bottom) throughout tidal waters of the Chesapeake Bay and its tributaries. Visual inspection of the maps produced by the Du and Shen model implied that salinity in some subestuaries was not well characterized by the model (e.g., the model predicted unexpectedly high salinity in the central portion of a subestuary). These cases were replaced with values obtained by linear interpolation between adjacent values. For simplicity, we first used depth-averaged mean salinity at each location. However, vertical salinity profiles in Chesapeake Bay are tidally and seasonally stratified with heavier, saltier waters near the bottom, and lighter, fresher water near the surface. Therefore, we also used surface salinity to predict habitat suitability at each location throughout the Chesapeake Bay. Surface salinity was defined as the mean of the predicted salinities in the top 1 m of the water column in each location; when salinity predictions were not available for the top 1 m, we used the predicted salinity of the topmost layer as the surface layer (0.002% of the cases; in these cases, the maximum depth of the topmost layer was 1.8 m). The predicted survival probability was mapped to mean or surface salinity conditions of Chesapeake Bay to obtain spatially-explicit, but static, representations of habitat suitability for blue catfish throughout the Chesapeake Bay and its subestuaries during spring (April) and fall (October) in years with average (2012), above average (2011) and below average (2009) freshwater discharge rates (“average”, “wet” and “dry” years, respectively). For comparison, the annual mean freshwater discharge rates into the Chesapeake Bay during 2009, 2011 and 2012 are estimated to be 1795, 3200 and 2265 m^3^s^-1^ respectively; the mean freshwater discharge rates during dry fall (October 2009) and wet spring (April 2011) were respectively 1418 and 7419 m^-3^s^-1^; a 5.2-fold difference in discharge rates (data from https://md.water.usgs.gov/waterdata/chesinflow/wy/).

## Results

### Distribution of blue catfish in Chesapeake Bay subestuaries

**Fig 4:**
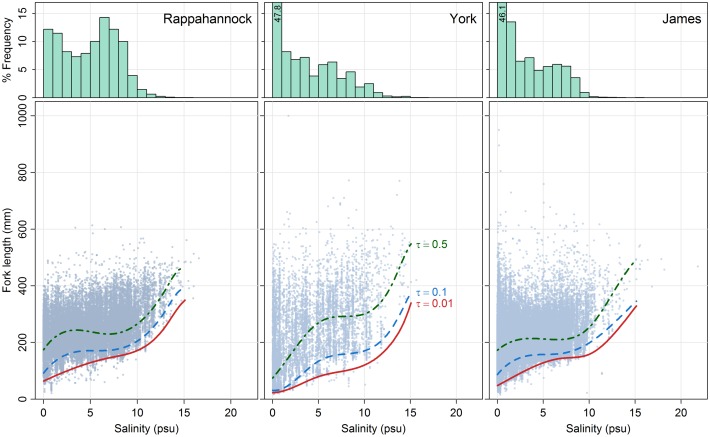
Bottom salinity and fork length of invasive blue catfish captured from the Rappahannock, York and James river subestuaries of the Chesapeake Bay during 1975–2017 by the VIMS juvenile fish trawl survey. The quantile regression splines for salinity ≤ 15 psu are shown for three quantiles (ε = {0.01, 0.1, 0.5}). Note that the y axis of the histogram is truncated for the York and James rivers; the percent of blue catfish captured at salinities < 1 psu in the York (47.8%) and James (46.1%) rivers are indicated in the figure.

A total of 178,611 blue catfish was collected from the Rappahannock (*n* = 76,322), York (*n* = 10,536) and James (*n* = 91,753) river subestuaries between 1975 and 2017 (S1 Dataset). Overall, 31.7% of blue catfish were collected from waters with salinity < 1 psu, and 98.6% were collected from waters with salinity < 10 psu, although subestuary-specific differences occurred (Fig 4). The highest salinity where blue catfish were collected was 21.8 psu in the James River. Quantile regression splines for each subestuary indicated an increase in size of fish with increasing salinity, such that individuals < 200 mm FL were primarily limited to salinities < 10 psu (Fig 4).

### Salinity tolerance

Mean body condition of blue catfish decreased systematically with increasing salinity (BIC for full model = -407.2; BIC for null model = -405.8); more specifically, the predicted mean Fulton’s K decreased from 1.06 to 0.98 (fraction change = 7.3%) when salinity increased from 0 psu (freshwater) to 10 psu. In contrast, the mean size of blue catfish did not differ among the salinity levels used in the experiment (BIC for full model = 1645; BIC for null model = 1638.9), suggesting that comparisons among salinity treatments were not biased by size differences.

**Fig 5:**
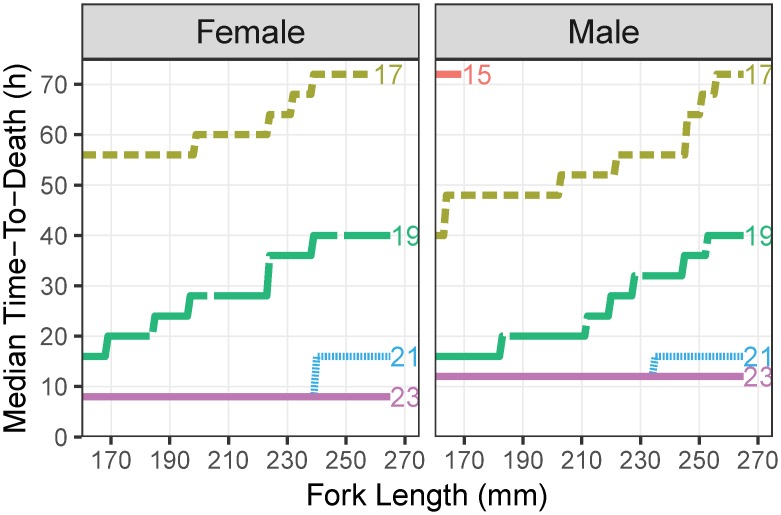
Median time-to-death (h) for male and female blue catfish at various salinities predicted by the stratified Cox proportional hazards model. Numbers at the end of the lines represent salinities in practical salinity units (psu). Figure available in color online.

Salinity had a negative effect on the time-to-death of blue catfish; a 1 psu increase in salinity increased the hazard (risk of death) by a factor of 9 (Table 1; Fig 5). However, the negative effects of salinity were lower for fish that were larger or had better body condition; larger fish with better body condition had a lower risk of dying (Table 1; Fig 5). Males and females had different baseline hazard rates such that the risk of death varied differently over time for male and female blue catfish.

**Table 1:** Parameter estimates for the stratified Cox proportional hazards model fit to the time-to death data from the toxicity test on blue catfish. CL = 95% confidence limit.

Variables	Estimate	Hazard Rate	Lower CL	Upper CL
Salinity	2.20	9.03	6.07	13.43
Fork length	-0.04	0.96	0.95	0.97
Condition	-5.93	0.002	<0.001	0.11

**Fig 6:**
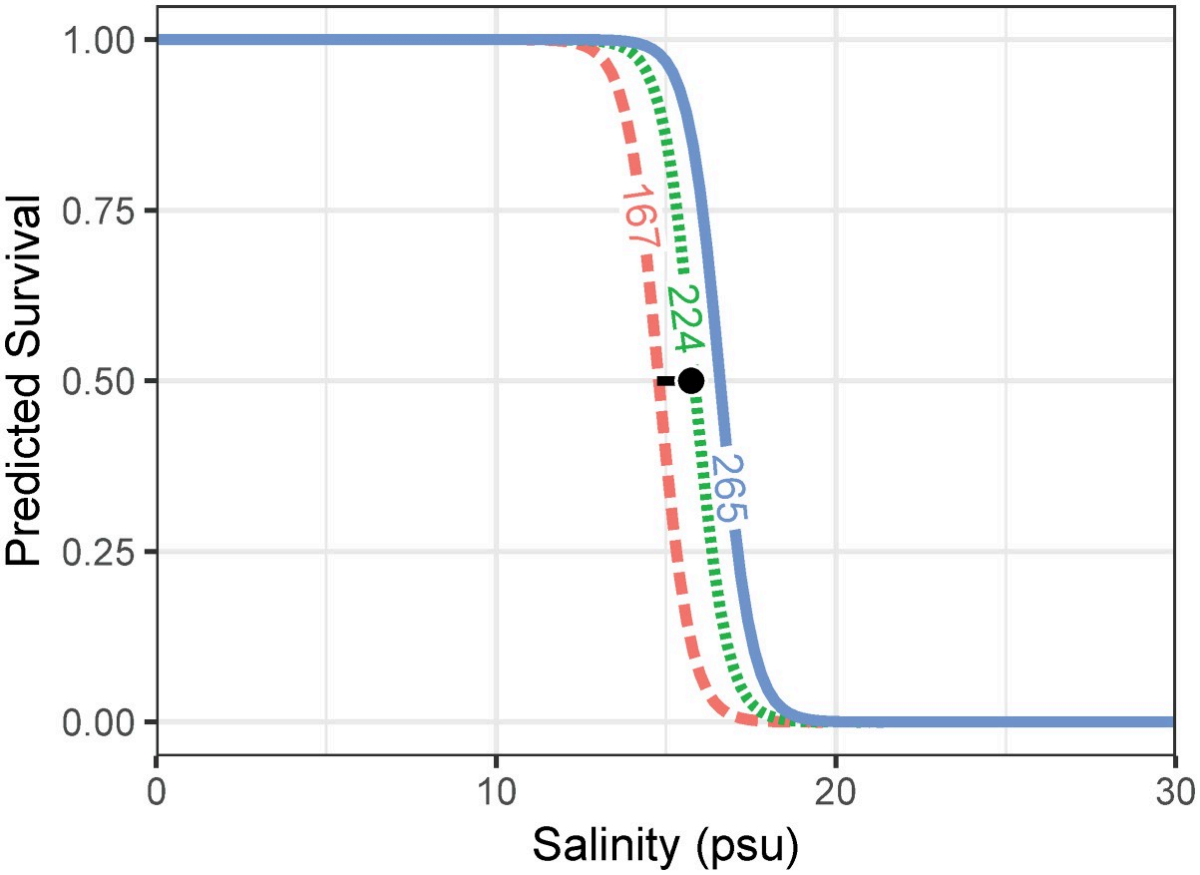
Predicted survival of blue catfish based on Firth Logistic regression fit to data from the 72-hour salinity tolerance experiment. The point and the bar correspond to the predicted salinity at 50% mortality (LC_50_) and the corresponding 95% confidence interval based on the logistic regression. Numbers along the line represent the minimum, median and maximum length (mm) of blue catfish used in this study.

None of the experimental blue catfish died at salinities ≤ 13 psu, and all blue catfish died at salinities ≥ 17 psu (S1 Dataset). At 16 psu, 11 of 30 experimental blue catfish (36.7%) died before the end of the experiment. The effects of salinity, fish length, condition and sex on whether the fish was alive at the end of 72-hours were analyzed with Firth’s logistic regression. The two most parsimonious models, accounting for total BIC weight of 0.89, were averaged to provide model-averaged parameter estimates for the effect of salinity and fish length on fate of the fish (Table 2). The odds of survival decreased by 88% with a 1 psu increase in salinity (odds ratio: 0.12; 95% confidence interval [CI]: 0.03–0.53) but increased by 5% with a 1 mm increase in fork length (odds ratio: 1.05; CI: 1.00–1.09; Table 3). The 72-hour LC_50_ from the averaged model was 15.7 psu (CI: 14.7–16.1; Fig 6). In comparison, the corresponding LC_50_ based on the Spearman-Karber method was 15.2 psu (CI: 14.8–15.7).

**Table 2:** Bayesian Information Criterion (BIC), ΔBIC, number of parameters and BIC weight for Firth logistic regression models fitted to describe the 72-hour mortality of blue catfish exposed to various salinities. The two most parsimonious models, highlighted in bold, were averaged to determine the final model. FL = Fork length.

Variables included	BIC	No. pars.	ΔBIC	Weight
**Salinity, FL**	**50.8**	**3**	**0**	**0.54**
**Salinity**	**51.7**	**2**	**0.9**	**0.35**
Salinity, FL, Sex	54.8	4	4	0.07
Salinity, FL, Condition	56.2	4	5.4	0.04
Salinity, FL, Condition, Sex	60	5	9.2	0.01
Salinity, FL, Condition, Sex, Aquarium	147.1	21	96.3	0
Salinity, FL, Sex, Aquarium	144.2	20	93.4	0
Salinity, FL, Condition, Aquarium	144.1	20	93.3	0

**Table 3:** Parameter estimates for the most parsimonious Firth logistic regression model to describe the 72-hour mortality of blue catfish exposed to various salinities. CL = 95% confidence limit.

Parameters	Estimate	Odds Ratio	Lower CL	Upper CL
Intercept	25.02	7.36×10^10^	1.53	3.5×10^21^
Salinity	-2.14	0.12	0.03	0.53
Fork length	0.04	1.05	1.00	1.09

### Habitat suitability mapping

**Fig 7:**
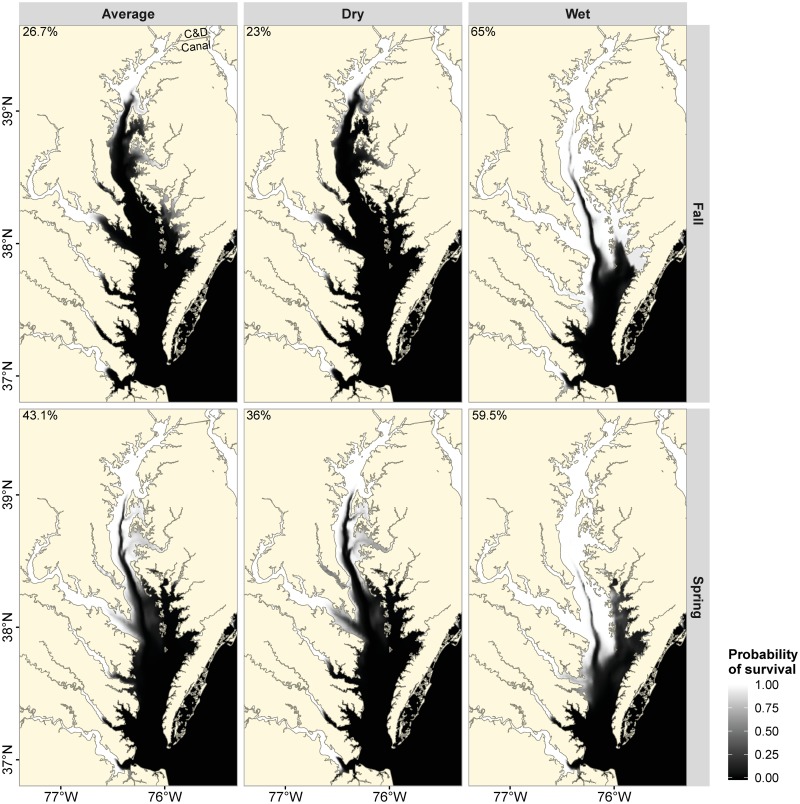
Spatially explicit probabilities of survival (72-hour) for a 224 mm blue catfish (median length in the salinity tolerance experiment) throughout the Chesapeake Bay based on vertically-averaged salinities in spring (April) and fall (October) during average (2012), dry (2009) and wet (2011) years. Number at the top left corner of each panel denotes the percent area of the Chesapeake Bay where predicted probability of survival for blue catfish was greater than 0.8. Note that the probability of survival was nearly 1 in the Chesapeake and Delaware Canal (C&D Canal).

**Fig 8:**
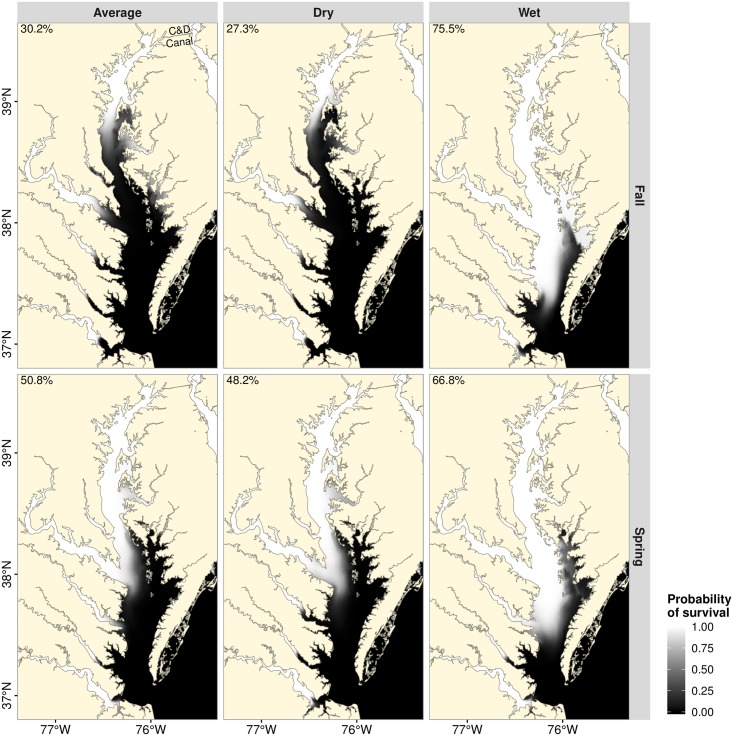
Spatially explicit probabilities of survival for a 224 mm blue catfish throughout the Chesapeake Bay based on surface salinities. See Fig 7 for additional details.

Static representations of Chesapeake Bay during typical average, dry and wet months show considerable spatial variation in average predicted salinity (Fig 2). In addition to the north-south gradient in the mainstem of Chesapeake Bay and the headwater-mouth gradient in the tributaries, there are also seasonal and inter-annual differences in salinity gradients (Fig 2). Vertically-averaged salinity in spring was lower than that in fall during dry and average precipitation years; in wet years, there was a considerable reduction in salinity during both spring and fall (Fig 2). Vertical, seasonal and annual variability in salinity resulted in marked variation in habitat suitability throughout the Chesapeake Bay. As expected, the proportion of suitable habitat was highest towards the headwater of the tributaries and at the head of the Chesapeake Bay (Figs 7 and 8). In addition, larger areas of Chesapeake Bay became habitable during wet months compared with dry months (Figs 7 and 8). For example, under the vertically-averaged salinity scenario in fall, the predicted probability of survival for a 224 mm FL blue catfish during average conditions exceeded 0.8 in only about 26.7% of Chesapeake Bay habitats, but the proportion of habitable area increased to 65% during wet conditions (Fig 7). Corresponding predicted proportions of habitable areas increased further to 30.2% and 75.5% respectively if only the surface layers are considered (Fig 8). Of note here is that the probability of survival was near 1.0 in the Elk River regardless of the discharge scenario, suggesting that salinity conditions in the Elk River could facilitate blue catfish dispersal into Delaware Bay via the Chesapeake and Delaware Canal (Figs 2, 7 and 8).

## Discussion

Blue catfish survived short-term (72-hr) exposure to mesohaline waters (< 15 psu), indicating that this species has the potential to survive in most downstream areas of major rivers entering Chesapeake Bay, and to use the mainstem of the upper Bay for movement into other subestuaries in Maryland and into Delaware Bay. Large (> 200 mm FL) individuals in particular are more tolerant of salinities > 10 psu than smaller, immature fish, and thus have a greater ability to use mesohaline and polyhaline habitats and invade additional areas throughout the Chesapeake Bay. Large individuals during wet months may exhibit jump dispersal, which is characterized by occasional long-distance movements; such movements are likely to increase the rate of spatial expansion [29] and the probability of regional persistence [30] of non-native blue catfish in the Chesapeake Bay region.

The salinity tolerance of blue catfish (LC_50_ = 15.7 psu) was higher than that of many freshwater fishes such as the percichthyid *Nannatherina balstoni* (LC_50_ = 8.2 psu; 72 hrs; [31]), and eastern mosquitofish *Gambusia holbrooki* (11.5 psu; 96 hrs; [32]), including some species of catfishes such as South American sailfin catfish *Pterygoplichthys* spp. (10.6 psu; 96 hrs; [33]) and African sharptooth catfish *Clarias lazera* (10.5 psu; 72 hrs; calculated based on [34]). The observed LC_50_ is, however, comparable to that of other members of the family Ictaluridae: flathead catfish *Pylodictis olivaris* (14.5–15.8 psu [35]); black bullhead *Ameiurus melas* (13.8 psu [36]); white catfish (14.0 psu [37]); channel catfish *Ictalurus punctatus* (12.0–15.5 psu [14,36]). Kendall and Schwartz [37] hypothesized that the relatively less permeable integument of catfishes might allow them to tolerate greater osmotic stress resulting in the somewhat high salinity tolerance of ictalurid catfishes. There is, however, little empirical support for this hypothesis because a majority of the water or ion exchange in fishes occurs across the gills but not the integument [4].

Although our results are consistent with previous studies with other ictalurid fishes, they may have been affected by our experimental procedures. First, the gradual increase in salinity we employed is unlike protocols that include abrupt changes from freshwater to the target salinity, or those that gradually increase salinity over several days [35]. In general, gradual increases in salinity result in estimates of salinity tolerances that are higher than those observed under abrupt changes (e.g., [34,35,38]). Second, the source or type of salt used in salinity tolerance experiments also varies among studies. These have included synthetic sea salt (Instant Ocean^®^; [35]), sodium chloride solutions (NaCl which comprises about 85% of the salts in seawater; e.g., [35]), diluted seawater (e.g., [37]) and diluted water from brine ponds (e.g., [36]). These differences have been shown to affect the measured salinity tolerances. For example, Bringolf et al. [35] observed that the 72-hr LC_50_ for juvenile flathead catfish was significantly lower when fish were exposed to NaCl solutions (10.0 psu) than when fish were exposed to synthetic seawater (14.5 psu). We used Instant Ocean^®^ because of the compositional resemblance of the resulting solution to natural seawater. Instant Ocean^®^ was supplemented by water from the York River to ensure that any trace elements or compounds not available in Instant Ocean^®^ would be provided by York River water. Third, different life stages and sizes of fish affect the determination of salinity tolerance. As we demonstrate, larger blue catfish have better osmoregulatory abilities compared with smaller individuals. This finding is consistent with studies on a wide range of freshwater fishes [32,39–41]. Such observations might result from size- and age-dependent changes in allometric scaling of body size, and development of endocrine and ionoregulatory pathways [41]. Compared with larger individuals, smaller fish have higher weight-specific aerobic metabolic rates and higher gill surface area to body mass ratios [42]. The result is that smaller individuals have higher rates of passive ion and water exchange per unit body mass which must be compensated by higher rates of active ion exchange in the gill and gut. Finally, the time of removal of moribund fish may also have affected our estimate of time-to-death, which was accurate to ± 4 hours.

The relatively high salinity tolerance we observed in blue catfish may have resulted, at least in part, from the acclimation of fish at 2 psu for 2 weeks. Hyperosmotic abilities may be upregulated after acclimation to low or moderate salinity conditions, as reported for anadromous fishes such as the Gulf sturgeon *Acipenser oxyrhynchus desotoi* [43], white sturgeon *Acipenser transmontanus* [44] and various salmonid species [45]. Such mechanisms may be active in non-anadromous fishes as well, because salinity tolerance is likely a conserved trait [12]. The acclimation protocol we followed with blue catfish may have led to the upregulation of hyperosmotic abilities in these fish, and thus to increased salinity tolerance. In addition, blue catfish in the James River subestuary are regularly exposed to low to moderate salinities. Therefore, osmotic abilities of blue catfish undergoing dispersal events, particularly those at the leading edge of the invasion, would likely be upregulated, and thus, the fish from the James River subestuary may have had an increased physiological ability to use brackish waters. Likewise, the salinity tolerance of blue catfish that were hatched in brackish conditions may be higher than that of fish hatched in freshwater, as demonstrated for Nile tilapia *Oreochromis niloticus* [46]. Such upregulation of salinity tolerance at the egg stage suggests that salinity tolerance of blue catfish may increase as the population expands into estuarine waters; upregulation may allow blue catfish to exploit a large portion of the Chesapeake Bay, possibly even exceeding the exploitable areas predicted here. Further, salinity tolerance of fishes can vary based on their genetic makeup and geographic distribution [40]; therefore, future research should compare salinity tolerance of different blue catfish populations within the Chesapeake Bay region.

Blue catfish exposed to high salinities had the lowest mean body condition indices, suggesting that fish may lose body mass under these conditions. We did not measure weight of fish before the salinity trials commenced but presume that the mean body mass of the blue catfish assigned to different salinity levels were similar because individuals were assigned randomly to salinity treatments. Reduction in body mass has been observed in other fishes such as California halibut *Paralichthys californicus* [47] and shortnose sturgeon *Acipenser brevirostrum* [48] when exposed to elevated salinities. Such decreases in body mass and condition are likely caused by a reduction in muscle water content [48,49]. Loss of muscle water content is often accompanied by increased plasma osmolality and indicates a breakdown of osmoregulatory abilities [49]; a critical level of water loss at high salinities could lead to mortality [48].

Our predictions of suitable habitats for blue catfish in the Chesapeake Bay are likely conservative and may be only relevant for the size range we studied. We gradually increased salinity over seven hours and exposure to the target salinity was 72 hours. In the wild, the rate of increase in salinity could be slower. The effects of temperature on salinity tolerance of blue catfish, though not investigated in this study, could also influence the predictions in this study. For example, when exposed to high salinity conditions, individuals of the tropical freshwater fish oscar *Astronotus ocellatus* survived longer at 28°C than at 18°C [50]. If such patterns hold for blue catfish, then warming water temperatures due to climate change would favor survival and dispersal of this species. This also highlights the potentially counteracting effects of high precipitation and freshwater influx on dispersal of this fish. Whereas high freshwater influx from headwaters into the subestuaries decreases salinity and positively influences the likelihood of dispersal, such events are typically accompanied by cooler temperatures, which somewhat offset the positive effect of lower salinities. However, the positive effect of decreased salinity likely outweighs the negative effects of decreased temperature. Future research should explicitly study the relative influences of temperature and salinity on survival and dispersal of blue catfish. In addition to mortality, high salinities may have sublethal effects on growth [1], reproduction [39] and metabolic rates [1] of fishes. Therefore, salinities > 9 psu may further limit the long-term occupation of estuarine habitats by blue catfish. Sublethal effects may explain why relatively few blue catfish have been consistently captured at salinities > 9 psu in the Rappahannock, York and James rivers. Sublethal effects of increased salinity should be investigated to obtain better predictions of blue catfish range expansion in the Chesapeake Bay.

The ability of blue catfish to use estuarine waters to expand in range and colonize novel habitats throughout the Chesapeake Bay region is aided by the most energetically efficient mode of transportation available to animals, swimming [4]. With a sustained swimming speed of 30 cm s^-1^ [51], in 72 hours a 250 mm blue catfish would be able to move 77.8 km, which is greater than the maximum width of Chesapeake Bay (48 km). A mark-recapture study on blue catfish in the Potomac River in the Chesapeake Bay has shown that blue catfish are capable of such long-distance movements [52]. Telemetry tracking of this species using acoustic tags equipped with depth sensors is needed to elucidate the effects of seasonal distribution and vertical stratification in salinity on size-specific habitat use and dispersal of blue catfish. To this end, we showed that low salinity surface waters can provide suitable habitats for blue catfish dispersal. Such behavior has been observed in the freshwater pikeperch *Sander lucioperca*, which exhibits increased swimming and vertical movement within the water column at salinities greater than 12.5 psu [19]. Overall, we conclude that blue catfish have the potential to expand to most subestuaries on both sides of the Chesapeake Bay, and also to the Delaware Bay via the Chesapeake and Delaware Canal. The role of the Canal as a two-way bridge for exchange of fishes between the Chesapeake and Delaware bays was highlighted by Brown et al. [19], who proposed that flathead catfish—another introduced ictalurid catfish—may have dispersed from the Delaware Bay (where they were introduced) into the Susquehanna River (a river entering the northern end of Chesapeake Bay, see Fig 1) drainage via this route. We postulate that some subestuaries in the Chesapeake Bay region are less likely to supply fish for cross-estuary movements. In particular, blue catfish in the James and York river subestuaries are less likely than those from other tributaries to disperse and colonize adjacent systems because of the considerably high salinities at the mouths of these subestuaries.

Despite their presence in the Chesapeake Bay region since the 1970s, blue catfish have not yet invaded some of the Chesapeake Bay subestuaries or the Delaware Bay. We postulate that this is because dispersal of blue catfish from one subestuary to another is largely restricted by salinity conditions in the Chesapeake Bay, and most of the inter-subestuary dispersal occurs only during high precipitation events when salinity declines. In the future, the frequency of extreme wet events is expected to increase, resulting in fluctuating salinity distributions throughout the Chesapeake Bay [13]. Such events will likely facilitate dispersal of blue catfish in the Chesapeake Bay. Tropical storms may also affect the dispersal of blue catfish. The short-term pulse in salinity (maximum of 10–15 psu for 12–36 hours) at oligohaline reaches of Chesapeake Bay during tropical storm events [53] is likely insufficient to cause mass mortality of blue catfish. However, the inundation of coastal lands during such storms and the subsequent declines in salinity throughout the lower portions of the Bay could provide opportunities for further range expansion. Such conditions were observed during tropical cyclone Isabel in 2003 [53].

Our findings highlight that resource managers and conservationists should be concerned about the potential for blue catfish to continue their range expansion in the rivers draining into the Chesapeake Bay and to impact negatively the native invertebrate and fish species of commercial, recreational and cultural value such as the blue crab *Callinectes sapidus* and Atlantic menhaden *Brevoortia tyrannus*, as well as species of conservation concern such as the catadromous American eel *Anguilla rostrata* and anadromous Atlantic sturgeon *Acipenser oxyrhynchus oxyrhynchus* (J. Watterson, pers. Comm., Naval Facilities Engineering Command Atlantic; [8,11]). Although diet studies of blue catfish in oligohaline habitats of Chesapeake Bay suggest relatively low predation rates on such species [11], similar studies have not been conducted in the mesohaline habitats. Because of the high population densities [9,54] and relatively large sizes of blue catfish in mesohaline areas, their overall impact on native species is likely to be substantial. More importantly, their expansion into the Delaware Bay would have a similar impact.

The development of spatio-temporally explicit management plans may assist in the management of blue catfish by limiting the range expansion of this species in the region. Because range expansion potential is maximized during wet months and years, increased monitoring of likely dispersal corridors during these periods may allow selective removal of blue catfish, and disruption of dispersal processes. Proactive prevention and early eradication of blue catfish in novel habitats is likely to be the best approach to minimizing the negative impacts of this invasive species.

## Supporting information

S1 Dataset Data on blue catfish captured from three Chesapeake Bay subestuaries (sheets SurveyData and SurveyMetadata) or used in salinity tolerance experiments (sheets ExperimentResults and ExperimentMetadata). (XLSX)
